# Joint Optimization of Receiver Placement and Illuminator Selection for a Multiband Passive Radar Network

**DOI:** 10.3390/s17061378

**Published:** 2017-06-14

**Authors:** Rui Xie, Xianrong Wan, Sheng Hong, Jianxin Yi

**Affiliations:** 1 Radio Detection Research Center, School of Electronic Information, Wuhan University, Wuhan 430072, China; xierui@whu.edu.cn (R.X.); jxyi@whu.edu.cn (J.Y.); 2 Cognitive Radio Sensor Networks Laboratory, School of Information Engineering, Nanchang University, Nanchang 330038, China; shenghong@ncu.edu.cn

**Keywords:** passive radar network, receiver placement, illuminator selection, partition *p*-center problem, set covering problem

## Abstract

The performance of a passive radar network can be greatly improved by an optimal radar network structure. Generally, radar network structure optimization consists of two aspects, namely the placement of receivers in suitable places and selection of appropriate illuminators. The present study investigates issues concerning the joint optimization of receiver placement and illuminator selection for a passive radar network. Firstly, the required radar cross section (RCS) for target detection is chosen as the performance metric, and the joint optimization model boils down to the partition *p*-center problem (PPCP). The PPCP is then solved by a proposed bisection algorithm. The key of the bisection algorithm lies in solving the partition set covering problem (PSCP), which can be solved by a hybrid algorithm developed by coupling the convex optimization with the greedy dropping algorithm. In the end, the performance of the proposed algorithm is validated via numerical simulations.

## 1. Introduction

In recent years, passive radars that utilize the broadcast, communication, radionavigation, or presence of non-cooperative radar signals as illuminators of opportunity have become research hotspots [1,2,3,4,5,6]. The radar netting strategy is gradually employed for passive radars to enhance their performance (such as coverage, positioning accuracy and tracking precision) [7,8,9,10,11,12]. Because the performance of a passive radar network (PRN) greatly depends on its geometric configurations [1,13,14,15,16,17,18,19], the topology optimization problem for a PRN has been widely researched.

There are a variety of evaluating indicators for the topology optimization of PRN, and the most important two are coverage and positioning accuracy. The previous literature has paid more attention to the positioning accuracy optimization of PRN [15,16,17,20]. In [15], the optimization model was converted into a knapsack problem. Then it was solved by a greedy algorithm. The effect of the initial value on algorithm performance was discussed. In [20] two transmitter subset selection strategies for the identification of optimal sets in FM-based PRNs were developed. The purpose was to minimize the number of selected transmitters for a predetermined positioning performance threshold or to minimize the positioning error with a given subset size of selected transmitters.

However, little work has been done to address the coverage evaluating indicator. The coverage defines how well the target of interest is monitored, which is a precondition for radar work. In a previous study [21], we preliminarily discussed issues concerning the coverage optimization and proposed a corresponding solving algorithm. This paper conducts further studies on this problem and considers possible improvements for receiver placement and illuminators selection based on the previous paper’s findings.

The importance of the receiver placement has been discussed in [21], hence we will focus on the importance of the illuminator selection here. The illuminator selection problem in a PRN should be considered from the uncontrollability of illuminator distributions and the advantages of multiband detection. Firstly, the illuminators of opportunity in the PRN are generally uncontrollable, and their distributions do not entirely aim towards radar detection. Therefore, different illuminators are selected for target detection in different regions. For example, two kinds of illuminators with varying frequencies and coverage areas are presently used in Wuhan, China, which are the China Mobile Multimedia Broadcasting (CMMB) and Digital Television Terrestrial Multimedia Broadcasting (DTMB) both in the ultrahigh frequency (UHF) band. The working frequency of the radar receiver is determined by the illuminators in different areas of interest. Secondly, multiband detection has significant advantages given that multiband radars can improve the detection sensitivity and extend the coverage area. A multiband detection network can be constructed to achieve the performance requirement, especially if greater surveillance ranges are required for target detection.

Therefore, the joint optimization of receiver placement and illuminators selection can be treated as a multiband PRN construction problem, and three fundamental questions should be considered:

(1) How many receivers should be placed?
(2) Where will the receivers be placed?
(3) Which frequency should be selected for each receiver?

By considering the questions (2) and (3) comprehensively, a joint optimization model for the multiband PRN is built with the criterion of the required RCS for target detection. In our optimization model, the total coverage is a combination of the multiple subareas because of the dissimilarities in different coverage areas of each individual illuminator. Therefore our optimization model is named a partition *p*-center problem (PPCP), which can be simplified into a *p*-center problem (PCP) when only one frequency and the first order coverage are considered. In the PPCP, the question (1) is regarded as a constraint. When this constraint is exchanged with the objective function of the PPCP, we can establish a partition set covering problem (PSCP), which also can be simplified into a set covering problem (SCP). The PSCP is conducive to solve the PPCP through the above conversion method. Therefore, both the PPCP and PSCP are concerned in this paper.

The PCP and SCP are combinatorial optimization problems, and are NP-complete [22]. The PCP is commonly used in national defense and military fields because it tries to optimize the worst conditions. The SCP is a kind of problem that minimizes the total number of receivers or the construction cost to satisfy the coverage requirements. These two problems are the basis for the study of the PPCP and PSCP, hence we will discuss the related research of these two problems respectively in the following content.

The PCP can be solved by heuristic and the exact algorithms. In [23], a tabu search and variable neighborhood search were used. In [24], the author summarized many heuristic algorithms, such as the greedy, interchange, alternate (Voronoi diagram) and neighborhood search algorithms. Based on these algorithms, a scatter search algorithm was proposed, which is a hybrid algorithm that was developed by combining with several heuristic algorithms. The simulation for this hybrid algorithm exhibited significant improvements in the global search performance [24]. Heuristic algorithms are fast in solving the PCP, however, the optimization results may be trapped within the local optimal solution if these algorithms are used alone. Therefore, an exact algorithm similar to the dichotomy method for solving the root value of an equation was proposed in [25,26]. This algorithm relies on iteratively solving a series of SCPs. At each iteration, it sets a threshold value to judge on the fulfillment of the constraints of PCP, and updates its lower and upper bounds in light of this information. The key idea of this algorithm will be adopt in our PPCP solution.

The SCP also includes both the heuristic and the exact algorithms. The heuristic algorithms include the greedy algorithm [27], ant colony algorithm [28] and local search [29]. A previous report has discussed the exact algorithm as it is represented by the branch and bound algorithms [30]. The SCP can also be solved by the convex programming technique. Convex optimization is a recently developed heuristic optimization algorithm [31]. Through convex relaxation, this heuristic algorithm constructs a convex problem or several sequential convex problems to approximate the original problem. By solving the relaxed convex problem, an approximate optimal solution to the original problem is obtained. Convex optimization is attractive for both computational complexity and convergence. Nevertheless, a crucial step for the convex optimization technique is to transform the non-convex model into convex model [32,33,34,35]. Therefore, the convex relaxation method is the focus of the study in solving the SCP.

The PPCP and PSCP models are different from the conventional PCP and SCP models because of different network patterns. In the conventional sensor network, each sensor node is capable of sensing, such that the well-known performance measures from experimental design are convex functions of the placement vector, as discussed in [31,32,33]. However, in the PRN, each receiver needs to combine with a transmitter to form a sensing unit. This will lead to a different performance measure and a different placement problem. Considering the multiband and the higher order coverage, the established optimization model will be difficult to solve directly by the existing convex optimization.

The first contribution of our research work lies in the establishment of the PPCP model. The PPCP model is more complex as compared to the simpler fundamental optimization problem, which only includes one frequency. Its complexity is derived from two aspects, i.e., the bistatic configuration and *K*-coverage requirements, which is defined as the discovery of a receiver placement mode such that every target in the region is covered by at least *K* amounts of transceiver pairs. The second contribution of our research work is a proposal of the solving algorithm for the PPCP model. A bisection algorithm to solve the PPCP is proposed based on the characteristics of the objective PPCP function. Since the bisection algorithm relies on the solutions of a series of PSCPs, the algorithm for solving the PSCP is the third contribution of our research work. This study consists of two aspects, namely the convex relaxation method for the PSCP and a hybrid algorithm developed by combining the convex optimization and the greedy dropping algorithm for solving the relaxed convex problem of the PSCP.

The rest of this paper is organized as follows: in Section 2, we introduce the joint optimization model for the placement of receivers and selection of illuminators in a passive radar system. In Section 3, we develop a bisection algorithm to solve the PPCP. A hybrid algorithm for solving the PSCP is then proposed in Section 4. The simulation results are demonstrated in Section 5. Finally, conclusions are drawn in Section 6.

## 2. Problem Description

### 2.1. Definitions

In a multiband radar, the transmitters may present as a single frequency network (SFN) structure, wherein multiple illuminators simultaneously work at the same frequency and transmit the same signal. The symbols of the scenario parameters are listed as follows:

N: Number of types of available illuminators;
$\Omega_{F}=\{f_{1},f_{2},\cdots ,f_{N}\}$: Frequency set;
$C_{n}$: Number of transmitter sites for each type of illuminator;
$\Omega_{Tn}=\{t_{n,1},t_{n,2},\cdots ,t_{n,C_{n}}\}$: Transmitter coordinate set for each type of illuminator;
$\Omega_{T}=\{\Omega_{T1},\Omega_{T2},\cdots ,\Omega_{TN}\}$: Total coordinates of the illuminators;
M: Number of the target;
$\Omega_{M}=\{s_{1},s_{2},\cdots ,s_{M}\}$: Target set;
J: Number of the optional receiver sites;
$\Omega_{OR}=\{r_{1},r_{2},\cdots ,r_{J}\}$: Optional receiver coordinate set.

The bistatic radar range equation for multiband radar can be expressed as:

(1) $$\begin{array}{cc} SNR_{m,n,i,j} & =P_{av,n,i}-L_{m,n,i}+\sigma_{rcs,m}-L_{m,n,j}\\ & +A_{r,n}+G_{n}-L_{s,n}-N_{0,n} \end{array}$$

where m indicates that the target follows $s_{m}\in \Omega_{M}$, n indicates that the working frequency follows. $f_{n}\in \Omega_{F}$, i indicates that the transmitter follows $t_{i}\in \Omega_{Tn}$, and j indicates that the receiver follows. $r_{j}\in \Omega_{OR}$. The meaning of the other variables is presented as follows:

$SNR_{m,n,n,j}$: Echo signal-to-noise ratio (SNR) of the target $s_{m}$ associated with the bistatic pair $\left(t_{i},r_{j}\right)$ and the working frequency $f_{n}$.
$P_{av,n,i}$: Effective isotropic radiated power of the transmitter $t_{i}$;
$L_{m,n,i}$: Propagation loss from the transmitter $t_{i}$ to the target $s_{m}$ at the working frequency $f_{n}$;
$\sigma_{rcs,m}$: RCS of the target $s_{m}$;
$L_{m,n,j}$: Propagation loss from the target $s_{m}$ to the receiver $r_{j}$ at the working frequency $f_{n}$;
$A_{r,n}$: Receiving antenna effective area at the working frequency $f_{n}$;
$G_{n}$: Processing gain at the working frequency $f_{n}$;
$L_{s,n}$: Hardware system loss at the working frequency $f_{n}$;
$N_{0,n}$: Noise power at the working frequency $f_{n}$.

According to (1), the required RCS for target detection can be expressed as:

(2) $$\sigma_{m,n,i,j}=L_{m,n,i}+L_{m,n,j}-G_{sys,n,i}$$

where $G_{sys,n,i}=P_{av,n,i}+A_{r,n}+G_{n}-L_{s,n}-N_{0,n}-SNR_{\min,n}$, $SNR_{\min,n}$ is the minimum detectable SNR which is related to the working frequency.

Therefore, we define the meaning of the coverage in a PRN: if the actual RCS is not less than the required RCS, then we infer that the target echo SNR is not less than $SNR_{\min,n}$ and claim that the target is covered.

In addition, the actual RCS has the characteristics of fluctuation and obeys some statistical probability distributions f(σ). Thus the target detection is probabilistic and the probability can be expressed as $\int_{\sigma_{m,n,i,j}}^{\infty }f(\sigma )d\sigma$. It can be deduced that the required RCS $\sigma_{m,n,i,j}$ is the upper quantile of the probability distribution and exhibits the confidence level of the target being covered.

### 2.2. Assumptions

Before introducing the optimization model, two basic assumptions that can simplify the model are described as follows:

(1) We assume the receiving antenna to be omnidirectional. In fact, the optimization model can be extended while the receiving antenna is directional. The extended method can refer to the multiband network in the following section.
(2) Similar statistical target characteristics are observed at different UHF band frequencies such that the required RCS for target detection can be used as the unified criterion for all frequencies. Otherwise, the evaluation criterion of the *K*-coverage probability should be calculated according to the RCS model and the required RCS.

### 2.3. Optimization Model

Undoubtedly, smaller required RCS, values result in higher probabilities of detecting the target. Therefore, the required RCS can be selected as the objective function for this study. To minimize the maximum value of the required RCS for all targets, namely optimizing the worst condition based on the fundamental PCP theory, the joint optimization problem is established in this subsection. The required RCS for each target can be given as:

(3) $$\sigma_{m,K}=\underset{f_{n}\in \Omega_{Fw}}{\min}\left(\underset{t_{i}\in \Omega_{Tn},r_{j}\in \Omega_{R}}{\min}\left(\Theta ,K_{n}\right)\right)$$

where min(Θ,K) is the *K*th minimum value in matrix Θ. When the number of elements in Θ is less than K, the value of min(x,K) is set to infinity. The structure of matrix Θ can be expressed as (4). It is a matrix with a size of $M\times (J\sum_{n=1}^{N}C_{n})$. Each row of the matrix Θ corresponds to a target and each column corresponds to a bistatic pair. In (3), $\underset{t_{i}\in \Omega_{Tn},r_{j}\in \Omega_{R}}{\min}\left(\Theta ,K_{n}\right)$ represents the required RCS for each frequency network. $\underset{f_{n}\in \Omega_{Fw}}{\min}$ indicates at least one frequency network is required to satisfy the *K*-coverage condition in the multiband PRN:

(4) $$\Theta =\left[\begin{array}{ccccccccc} \sigma_{1,1,1:C_{1},1} & \cdots & \sigma_{1,1,1:C_{1},J} & \cdots & \sigma_{1,n,i,j} & \cdots & \sigma_{1,N,1:C_{N},1} & \cdots & \sigma_{1,N,1:C_{N},J}\\ \sigma_{2,1,1:C_{1},1} & \cdots & \sigma_{2,1,1:C_{1},J} & \cdots & \sigma_{2,n,i,j} & \cdots & \sigma_{2,N,1:C_{N},1} & \cdots & \sigma_{2,N,1:C_{N},J}\\ \vdots & \ddots & \vdots & \ddots & \vdots & \ddots & \vdots & \ddots & \vdots \\ \sigma_{M,1,1:C_{1},1} & \cdots & \sigma_{M,1,1:C_{1},J} & \cdots & \sigma_{M,n,i,j} & \cdots & \sigma_{M,N,1:C_{N},1} & \cdots & \sigma_{M,N,1:C_{N},J} \end{array}\right]$$

In the area of interest, the required RCS for all targets, which represents the worst condition, can be formulated as:

(5) $$f(\Omega_{R},\Omega_{Fw})=\underset{s_{m}}{\max}\left(\sigma_{m,K}\right)$$

where $\Omega_{R}$ is a subset of $\Omega_{OR}$, and $\Omega_{Fw}$ is the corresponding operating frequency.

Based on the PCP theory, the optimization objective is to minimize this maximum value with P receivers configured. Then the optimization model in a multiband PRN can be formulated as:

(6) $$\begin{array}{l} \;\left(\Omega_{R}^{*},\Omega_{Fw}^{*}\right)=\underset{\Omega_{R}^{*},\Omega_{Fw}^{*}}{\mathrm{argmin}}\left(f(\Omega_{R},\Omega_{Fw})\right)\\ s.t.\;{\left\|\Omega_{R}\right\|}_{0}=P \end{array}$$

where ${\left\|\Omega_{R}\right\|}_{0}$ denotes the number of elements in $\Omega_{R}$.

### 2.4. Standard Form of Optimization Model

The standard PCP model consists of the standard inputs, the standard outputs and five kinds of constraints [24]. Its inputs include the cost matrix and the number of facilities to locate. The outputs consist of the placement matrix, the assignment matrix and the maximum distance between a demand node and the nearest facility. Its constraints are the maximum distance constraint, the assignment variables constraint, the placement variables constraint, the variables relationship constraint and the integrality constraint.

The standard form of the model (6) will be illustrated in this subsection according to the standard PCP model. Firstly, the cost matrix, which is one of the model inputs, has been set up as (4). Subsequently, we define the outputs of the model. A joint placement matrix U is defined as:

(7) $$U={\left[u_{1,1},u_{1,2},\cdots ,u_{1,J},u_{2,1},u_{2,2},\cdots ,u_{2,J},\cdots ,u_{n,j},\cdots u_{N,1},u_{N,2},\cdots ,u_{N,J}\right]}^{T}$$

where the superscript T denotes the transpose of a matrix. The placement variables $u_{n,j}$ used in our problem are defined as:

$$u_{n,j}=\left\{ \begin{array}{l} 1\;\mathrm{If}\;a\;\mathrm{receiver}\;\mathrm{is}\;\mathrm{located}\;\mathrm{at}\;r_{j},\;\mathrm{and}\;\mathrm{works}\;\mathrm{at}\;\mathrm{frequency}\;f_{n},\\ 0\;\mathrm{Otherwise}. \end{array}\right.$$

The placement variables ($u_{q}$) are the variables of the optimization problem, while $v_{m,q^{\prime }}$ signify the assignment variables and are defined as:

$$v_{m,q^{\prime }}=\left\{ \begin{array}{l} 1\;\mathrm{If}\;\mathrm{the}\;\mathrm{target}\;s_{m}\;\mathrm{is}\;\mathrm{assigned}\;\mathrm{to}\;\mathrm{the}\;q^{\prime }\mathrm{th}\;\mathrm{transceiver}\;\mathrm{pair},\\ 0\;\mathrm{Otherwise}. \end{array}\right.$$

The values of each subscript are:

$$\begin{array}{l} m=1,2,\cdots ,M\\ q=1,2,\cdots ,NJ\\ q^{\prime }=1,2,\cdots ,J\sum_{n=1}^{N}C_{n} \end{array}$$

The relationship between n and q can be formulated as n=floor((q−1)/J)+1, where floor(⋅) is the rounding down operation. Aiming at the variables relationship constraint, we should pay attention to the conversion relationship between the placement matrix and the feasible bistatic pairs. Thus a transform matrix is present as:

(8) $$T={\left[\begin{array}{ccccccc} E_{C_{1},1} & & & & & & \\ & \ddots & & & & & \\ & & E_{C_{1},1} & & & & \\ & & & \ddots & & & \\ & & & & E_{C_{N},1} & & \\ & & & & & \ddots & \\ & & & & & & E_{C_{N},1} \end{array}\right]}_{\left(J\sum_{n=1}^{N}C_{n})\times (NJ\right)}$$

where $E_{C_{n},1}$ is a matrix of ones with a size of $C_{n}\times 1$, and T is a matrix with a size of $\left(J\sum_{n=1}^{N}C_{n})\times (NJ\right)$.

When considering the assignment variables constraint, we must count the number of bistatic pairs associated with the assessment of the *K*-coverage condition in each frequency network. The statistical matrix can be expressed as:

(9) S=[EJ,1    EJ,1    ⋱    EJ,1](NJ)×N

where $E_{J,1}$ is a matrix of ones with a size of J×1, and S is a matrix with a size of (NJ)×N.

Let Q=TS, such that the standard *p*-center form of the model (6) can be expressed as:

(10a) min w

(10b) $$s.t.\;\Theta_{m,q^{\prime }}v_{m,q^{\prime }}\leq wv_{m,q^{\prime }}$$

(10c) $$v_{m,q^{\prime }}\leq \sum_{q}\left(T_{q^{\prime },q}u_{q}\right)$$

(10d) $$\underset{n}{\max}\left\{\sum_{q^{\prime }}\left(v_{m,q^{\prime }}Q_{q^{\prime },n}\right)-K_{n}\right\}=0$$

(10e) $$\sum_{q}u_{q}=P$$

(10f) $$v_{m,q^{\prime }},u_{q}\in \{0,1\}$$

In (10a), w is the objective function value. The meaning of each constraint in (10) can be described as follows: constraint (10c) prevents the assignment of a target to a bistatic pair, which generally does not work. Constraint (10d) requires all targets to satisfy the *K*-coverage condition in at least one frequency network. A total of P receivers must be configured, which is modeled by constraint (10e). Models in (10a) to (10f) can be boiled down to the PPCP. The reason is that the max function in constraints (10d) has the characteristic of the subsection, thereby partitioning the coverage area of each frequency network.

The PPCP can be simplified into the PCP. When $K_{n}=1$, for $v_{m,q^{\prime }}\in \{0,1\}$, constraints (10d) can be expressed as $\underset{n}{\max}\left\{\sum_{q^{\prime }}\left(v_{m,q^{\prime }}Q_{q^{\prime },n}\right)\right\}=1$. If N=1, then $\sum_{q^{\prime }}v_{m,q^{\prime }}=1$ can be derived from constraints (10d), and these are the assignment variables constraint in the traditional PCP. On the other hand, the structure of Θ can be further simplified when $K_{n}=1$. For each optional receiver and each working frequency, only the minimum required RCS of the corresponding feasible bistatic pairs must be given, thereby resulting in equal number of q and $q^{\prime }$ values and converting the transform matrix T into an identity matrix, which allows constraints (10c) to be converted into $v_{m,q}\leq u_{q}$. These constraints describe the relationship between the assignment variables and the placement variables in the traditional PCP. The abovementioned statements verify the PPCP as an upgraded problem of the PCP. In this paper, we will mainly focus on solving the PPCP to obtain a joint optimization allocation scheme for receiver placement and illuminator selection in a multiband PRN.

## 3. The Proposed Algorithm for Solving the PPCP

In this section, a bisection algorithm was proposed to solve the PPCP, thereby requiring the characteristic of the objective function to first be analyzed.

**Proposition** **1.** *Assume that the optimal objective function value in (10) is* $w^{*}(p)$ *with* p *receivers configured, thus* $w^{*}(p)$ *decreases with the increase of* p.

**Proof.**  Assume the corresponding placement matrix of $w^{*}(p)$ is $U^{*}(p)$. The set of receivers to be configured is $S_{o}^{*}(p)=\{p|U^{*}(p)=1\}$, and $S_{uo}^{*}(p)=\{p|U^{*}(p)=0\}$ represents the set of receivers that don’t open. Using $S_{o}^{*}(p)$, we constructed the solution $S_{o}(p+1|p)$ and the corresponding objective function value w(p+1|p). The construction method of $S_{o}(p+1|p)$ is $S_{o}(p+1|p)=S_{o}^{*}(p)\cup \{u_{nq}\}$, where $u_{nq}\in S_{uo}^{*}(p)$. Thus, $w(p+1|p)\geq w^{*}(p+1)$ can be inferred according to the principle of optimization.

On the other hand, ∀m, $w_{m}(p+1|p)=\min\left(w_{m}^{*}(p),\Theta_{m,uq^{\prime }}\right)\leq w_{m}^{*}(p)$, where $\Theta_{m,uq^{\prime }}$ is the corresponding value of the new receiver in the matrix. Thus $w(p+1|p)=\underset{m}{\max}\left(w_{m}(p+1|p)\right)\leq \underset{m}{\max}\left(w_{m}^{*}(p)\right)=w^{*}(p)$. Comprehensively, we can be sure that $w^{*}(p)\geq w^{*}(p+1)$.

It is difficult for the condition of this inequality to occur given the equality mark, although exist a very special network topology described in the Appendix A. Thus, $w^{*}(p)>w^{*}(p+1)$ is established under ordinary circumstances, wherein $w^{*}(p)$ decreases with an increase in p.  ☐

According to Proposition 1, the dichotomy used to solve the root of the equation is adopted to obtain the optimal objective function value, which requires reiterative guessing. At each iterative search, the guessed value must be adjusted to satisfy the constraints. In other words, the bisection algorithm is based on solving a series of PSCPs [26]. The PSCP can be expressed as:

(11a) $$\min\;{\left\|U\right\|}_{0}$$

(11b) $$s.t.\;\Theta_{m,q^{\prime }}v_{m,q^{\prime }}\leq w_{mid}v_{m,q^{\prime }}$$

(11c) $$v_{m,q^{\prime }}\leq \sum_{q}\left(T_{q^{\prime },q}u_{q}\right)$$

(11d) $$\underset{n}{\max}\left\{\sum_{q^{\prime }}\left(v_{m,q^{\prime }}Q_{q^{\prime },n}\right)-K_{n}\right\}=0$$

(11e) $$v_{m,q^{\prime }},u_{q}\in \{0,1\}$$

where ${\left\|U\right\|}_{0}$ denotes the number of nonzero entries in U. The bisection algorithm for solving the PPCP is given in Algorithm 1.

**Algorithm 1:** The bisection algorithm for solving the PPCP**Input**: The matrix Θ and the number of receivers that are to be configured P.**Output**: The optimal placement matrix $U_{opt}^{*}$ and the optimal objective function value $w_{opt}^{*}$.**Step 1**: Initialize the upper bound $u=w^{*}(1)$, the lower bound $l=w^{*}(NJ)$, and the iterative termination value ε.**Step 2**: If u−l≥ε then proceed to Step 3. Else, go to Step 4.**Step 3**: Set $w_{mid}=(u+l)/2$. Solve (11) and obtain the optimal solution $U_{\mathrm{PSCP}}$. If ${\left\|U_{\mathrm{PSCP}}\right\|}_{0}\leq P$ then set $u=w_{mid}$. Else, set $l=w_{mid}$. Return to Step 2.**Step 4**: Construct a set $W_{opt}$ that contains the possible optimal value according to u and l, such that $W_{opt}=\{\Theta_{m,q^{\prime }}|l\leq \Theta_{m,q^{\prime }}\leq u,\forall m,q^{\prime }\}$. Scan $w_{mid}\in W_{opt}$, solve (11) and obtain the optimal solution $U_{k}$ and the optimal objective function value $w_{k}$. Thus the output can be expressed as $\left(U_{opt}^{*},w_{opt}^{*}\right)=\{\left(U_{k},w_{k}\right)|{\left\|U_{k}\right\|}_{0}=P,w_{opt,k}=\underset{k}{\min}(w_{k})\}$.

When the upper and lower bounds are quite close, the convergence rate of the dichotomy is extremely slow. Because the optimal objective function value must exist in the matrix Θ, fewer elements are present in $W_{opt}$, while u and l are quite close. The optimal solution can be obtained through the enumeration method, whose idea is embodied in Step 4.

## 4. The Proposed Algorithm for Solving the PSCP

According to Algorithm 1, the key to solving (10) is to first solve (11). In this section, a hybrid algorithm is combined the convex optimization with the greedy dropping algorithm to solve the PSCP.

### 4.1. Model Conversion

Given the difficulty in directly solving the model, we propose two conversion methods to simplify the model. The conversion methods are introduced in this subsection. Firstly, a decision matrix Ψ is constructed to meet the constraints presented in (11b). Ψ as the same size as Θ. If $\Theta_{m,q^{\prime }}\leq w_{mid}$, then $\Psi_{m,q^{\prime }}=1$. Else, $\Psi_{m,q^{\prime }}=0$. Thus, the constraints in (11c) and (11d) can be expressed as:

(12) $$\underset{n}{\max}(\Psi Tdiag(U)S-K)\geq O_{M\times 1}$$

where $O_{M\times 1}$ is an all-zero matrix with a size of M×1. diag(⋅) denotes the diagonal matrix of a matrix. K is a required coverage order matrix, and can be expressed as $K=E_{M\times 1}\left[\begin{array}{cccc} K_{1} & K_{1} & \cdots & K_{N} \end{array}\right]$. $E_{M\times 1}$ is an all-one matrix with a M×1 size.

The model in (11) becomes:

(13a) $$\min\;{\left\|U\right\|}_{0}$$

(13b) $$s.t.\;\underset{n}{\max}(\Psi Tdiag(U)S-K)\geq O_{M\times 1}$$

(13c) $$U\in {\left\{0,1\right\}}^{NJ}$$

The constraint in (13b) can be expressed as another form:

(14) $$\underset{n}{\max}(\Psi Tdiag(U)Sdiag(K^{\prime }))\geq E_{M\times 1}$$

where $K^{\prime }={\left[1/K_{1},1/K_{2},\cdots ,1/K_{N}\right]}^{T}$.

The model in (13) is the result of the first conversion method such that iteratively solves (13) based on the reweighted process introduced in [35]. However, the solution of this model is not ideal for quite a few scenes. Thus, (13) must be further converted by the second conversion method.

**Figure 1:**
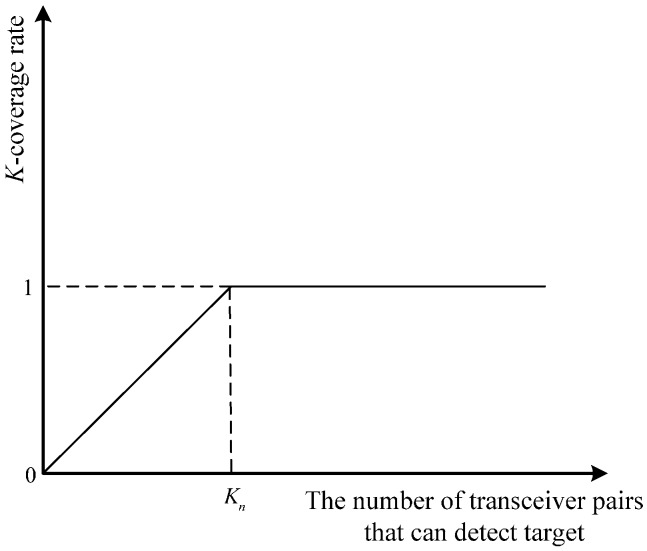
The curve of the *K*-coverage rate.

Let $\Psi^{\prime }=\Psi T$, where ${\Psi^{\prime }}_{m,q}>K_{n}$ indicates that the target can be covered by only one receiver. Its meaning is similar to that of ${\Psi^{\prime }}_{m,q}=K_{n}$. We construct a *K*-coverage rate matrix Φ in view of this case. The element of the matrix Φ can be expressed as $\Phi_{m,q}=\min\left({\Psi^{\prime }}_{m,q}/K_{n},1\right)$. The *K*-coverage rate represents the probability of satisfying the *K*-coverage condition. The curve of the *K*-coverage rate is shown in Figure 1.

Based on the *K*-coverage rate matrix, (13) becomes:

(15a) $$\min\;{\left\|U\right\|}_{0}$$

(15b) $$s.t.\;\underset{n}{\max}(\Phi diag(U)S)\geq E_{M\times 1}$$

(15c) $$U\in {\left\{0,1\right\}}^{NJ}$$

In the following subsection, we focus on solving (15), and (13) can be solved by the same method.

### 4.2. Sparsity-Enhancing Iterative Algorithm

In this subsection, we obtain an equivalent model of (15) using the convex relaxation method. The equivalent model is then solved by a sparsity enhancing iterative algorithm. The domain of the optimization variables and all the constraints in (15) are nonconvex. The convex relaxation method is described as follows. First, $U\in {\left\{0,1\right\}}^{NJ}$ is replaced by $U\in {\left[0,1\right]}^{NJ}$, where $U\in {\left[0,1\right]}^{NJ}$ means U is a (NJ)×1 vector for each element within the interval [0,1].

Next, the nonconvex constraint in (15b) is relaxed. The max function is replaced by the sum function, such that constraint in (15b) becomes:

(16) $$\Phi U\geq E_{M\times 1}$$

Therefore (15) can be relaxed as:

(17) $$\begin{array}{c} \min\;{\left\|U\right\|}_{0}\\ s.t.\;\left\{ \begin{array}{l} \Phi U\geq E_{M\times 1}\\ U\in {\left[0,1\right]}^{NJ} \end{array}\right. \end{array}$$

The sparsity enhancing reweighted $l_{1}$ minimization in [35] is an effective method for solving (17). This method is a recursive realization of the surrogate based on the sum of logarithms. The relaxation model of (17) can be expressed as:

(18) $$\begin{array}{c} \min\;V_{k}^{T}U\\ s.t.\;\left\{ \begin{array}{l} \Phi U\geq E_{M\times 1}\\ U\in {\left[0,1\right]}^{NJ} \end{array}\right. \end{array}$$

where $V_{k}$ is a weight matrix with a 1×(NJ) size, and k is the iteration counter as reweighted process is utilized. When k=0, the weighted matrix $V_{k}$ is initialized as an all-one matrix $E_{1\times (NJ)}$. When k≥1, $V_{k}$ is updated according to:

(19) $$V_{k}=\frac{1}{\delta +U_{k-1}^{T}}$$

where $U_{k}$ is the obtained optimal solution of the *k*th iteration. δ is a small positive value that prevents the denominator from becoming zero and enhances numerical stability. The model (18) presents a convex optimization model that can be solved within the polynomial time using interior-point methods. The iterative algorithm for solving (17) is summarized in Algorithm 2.

**Algorithm 2:** The sparsity enhancing iterative algorithm for solving (17)**Input**: The matrix Θ, $w_{mid}$, all $K_{n}$, the maximum number of iterations $k_{\max}$, δ and a small positive value γ.**Output**: The optimal placement matrix $U_{\mathrm{opt}\text{\_}\mathrm{cvx}}$.**Step 1**: Construct the *K*-coverage rate matrix Φ.**Step 2**: Initialize k=0, and $V_{0}=E_{1\times (NJ)}$.**Step 3**: Solve the model presented in (18).**Step 4**: Increase k. When k attains $k_{\max}$ or the maximum change in the entries of U is less than γ, terminate the iteration to generate the output. Otherwise, update the weight according to (19) and return to Step 3.

### 4.3. Iterative Varying Constraint Algorithm

The optimal solution in (17) can be obtained using Algorithm 2. However, this solution does not always satisfy the constraint in (15b) as it is directly replaced by the sum constraint. We further investigated the constraint in (15b), which can be expressed as:

(20) $$\Phi U\geq E_{M\times 1}+[\Phi U-\underset{n}{\max}(\Phi diag(U)S)]$$

If (20) is simplified as $\Phi U\geq E_{M\times 1}$, the constraint in (15b) may be not satisfied at some target points. An effective solution would be to adjust the constraint, and continue to solve the new model until (15b) is satisfied. This method is called the iterative varying constraint algorithm and is described as follows.

The iterative varying constraint algorithm is a fixed point iteration method. Assume that the probable optimal solution is $U_{p\text{\_}\mathrm{opt}}$, such that the new constraint that has a reinforcing effect on the original constraint is expressed as $\Phi U\geq E_{M\times 1}+[\Phi U_{p\text{\_}\mathrm{opt}}-\underset{n}{\max}(\Phi diag(U_{p\text{\_}\mathrm{opt}})S)]$. This iteration is predicted to reach equilibrium. At equilibrium, the solutions will no longer change, thus allowing the derivation of $\Phi U_{p\text{\_}\mathrm{opt}}\geq E_{M\times 1}+[\Phi U_{p\text{\_}\mathrm{opt}}-\underset{n}{\max}(\Phi diag(U_{p\text{\_}\mathrm{opt}})S)]$. The probable optimal solution at equilibrium satisfies the constraint in (15b).

Due to the complexity of the optimization function, the iterative varying constraint algorithm is not applicable for all cases. A special case is illustrated as follows. Assume that Φ=[1−α 1−α α 0], where 0<α≤1/3, and $S={\left[1\;1\;0\;0\;;\;0\;0\;1\;1\right]}^{T}$. The optimal solution $U={\left[0\;1\;1\;0\right]}^{T}$ can be obtained when the relaxed constraint is ΦU≥1. In the next iteration, the new relaxed constraint is ΦU≥1+α, and the corresponding optimal solution is $U={\left[1\;1\;0\;0\right]}^{T}$. Thereafter, the abovementioned constraints appear alternately. The optimal solution satisfies (13b) when the relaxed constraint is ΦU≥1+α.

Although the above example does not show the possibility of the iterative varying constraint algorithm converging to the fixed point, the algorithm is generally suitable for most actual situations. Monte Carlo (MC) simulation is used to obtain the probability of failure of the algorithm. The simulation results indicate a failure probability of less than 5% and show the applicability of the algorithm in most scenarios. The iterative varying constraint algorithm is summarized in Algorithm 3.

**Algorithm 3:** The iterative varying constraint algorithm for solving (15)**Input**: The matrix Θ, $w_{mid}$, all $k_{n}$, the maximum number of iterations $i_{\max}$, and a small positive value ε.**Output**: The optimal placement matrix $U_{\mathrm{opt}\text{\_}\mathrm{vc}}$.**Step 1**: Construct the *K*-coverage rate matrix Φ.**Step 2**: Initialize $i_{vc}=0$, and $E_{0}=E_{M\times 1}$.**Step 3**: Solve the following model using Algorithm 2.$$\begin{array}{c} \min\;{\left\|U\right\|}_{0}\\ s.t.\;\left\{ \begin{array}{l} \Phi U\geq E_{i_{vc}}\\ U\in {\left[0,1\right]}^{NJ} \end{array}\right. \end{array}$$**Step 4**: Increase $i_{vc}$. When $i_{vc}$ attains $i_{\max}$ or the maximum change of the entries of $E_{i_{vc}}$ is less than ε, terminate the iteration and output the solution. Otherwise, update $E_{i_{vc}}=E_{M\times 1}+[\Phi U_{\mathrm{opt}}^{\left(i_{vc}-1\right)}-\underset{n}{\max}(\Phi diag(U_{\mathrm{opt}}^{\left(i_{vc}-1\right)})S)]$, where $U_{\mathrm{opt}}^{\left(i_{vc}-1\right)}$ is the optimal solution in Step 3, then return to Step 3.

### 4.4. Greedy Dropping Algorithm

In the previous subsection, the probable optimal solution is obtained by using Algorithm 3. Although the solution satisfies (15b), it does not invariably satisfy (15c) because the solution may have some fractional values. This problem has been solved in previous literatures. In [35], a randomized rounding algorithm for optimizing the solution was proposed. Moreover, a previous report [16] adopted an ordered rounding algorithm. The present study proposes a greedy dropping method to optimize the solution.

We first construct a solution $U^{\prime }=\left\lceil U_{\mathrm{opt}\text{\_}\mathrm{vc}}\right\rceil$, where ⌈⋅⌉ is the rounding up operation. Given that the objective function of (15) is the least number of receivers, we continuously reduce the number of the receivers based on the solution $U^{\prime }$ until it reaches the minimum. The remaining nonzero placement variables construct the final solution. There are few nonzero placement variables in the solution $U^{\prime }$, thus the iterative process is terminated in a finite number of steps. The greedy dropping algorithm is summarized in Algorithm 4.

**Algorithm 4:** The greedy dropping algorithm**Input**: The solution $U_{\mathrm{opt}\text{\_}\mathrm{vc}}$, which is obtained using Algorithm 3.**Output**: The final optimal placement matrix $U_{\mathrm{opt}}$.**Step 1**: Construct the initial undetermined solution $U_{\mathrm{uc}}^{\left(0\right)}=\left\lceil U_{\mathrm{opt}\text{\_}\mathrm{vc}}\right\rceil$. Set flag=1. Initialize $i_{c}=0$.**Step 2**: If flag=1 then go to Step 3. Else, go to Step 4.**Step 3**: Increase $i_{c}$, and reset flag=0. Scan the entire solution of the last iteration. Set the nonzero placement variables in the solution to zero and construct a new solution $U_{\mathrm{uc}\text{\_}\mathrm{temp}}$. Test if $\underset{n}{\max}(\Phi diag(U)S)\geq E_{M\times 1}$ is satisfied. If satisfied, set flag=1 and add $U_{\mathrm{uc}\text{\_}\mathrm{temp}}$ to the solution of $U_{\mathrm{uc}}^{\left(i_{c}\right)}$. When the traversal is complete, return to Step 2.**Step 4**: Terminate the iteration. The final optimal solution is $U_{\mathrm{opt}}=U_{\mathrm{uc}}^{\left(i_{c}\right)}$. 

### 4.5. Analysis of Computation

In this subsection, we emphatically analyze the computation of the proposed algorithms. Assume that the iteration times of Algorithms 2 and 3 are $K_{2i}$ and $K_{3i}$, respectively. The complexity of the interior-point methods is $O\left(NJ\log(1/\varepsilon_{cvx})\right)$, where $\varepsilon_{cvx}$ is the required accuracy parameter. Thus the complexity of Algorithm 3 is $O\left(K_{2i}K_{3i}NJ\log(1/\varepsilon )\right)$. The computational complexity of Algorithm 4 can be neglected because it only requires some finite matrix multiplication and numerical comparison operations.

In Step 3 of Algorithm 1, the number of solving the PSCP is assumed to be $K_{1i}$. In Step 4 of Algorithm 1, the number of the elements in $W_{opt}$ is $K_{1e}$. In Step 4, it requires O(M(α+N)) time to compute the objective function value, where $\alpha =\sum_{n=1}^{N}C_{n}P\log_{2}(C_{n}P)$. The total time complexity for Algorithm 1 can be deduced as $O\left(\left(K_{1i}+K_{1e})K_{2i}K_{3i}NJ\log(1/\varepsilon )+K_{1e}M(\alpha +N\right)\right)$.

In contrast, the complexity of the substitution algorithm (SA) which is introduced in [24] is analyzed. In each iteration, it requires P(NJ−P) times to compute the objective function value. Assume that the iteration times of SA is $K_{SA}$, then the total time complexity of SA can be expressed as $O\left(K_{SA}P(NJ-P)M(\alpha +N)\right)$.

Normally, $K_{SA}P(NJ-P)M(\alpha +N)\;<\;(K_{1i}+K_{1e})K_{2i}K_{3i}NJ\log(1/\varepsilon )+K_{1e}M(\alpha +N)$, thus Algorithm 1 is more time consuming than SA. However, SA usually requires a host of simulations to achieve the global optimal solution.

## 5. Simulation Results

**Figure 2:**
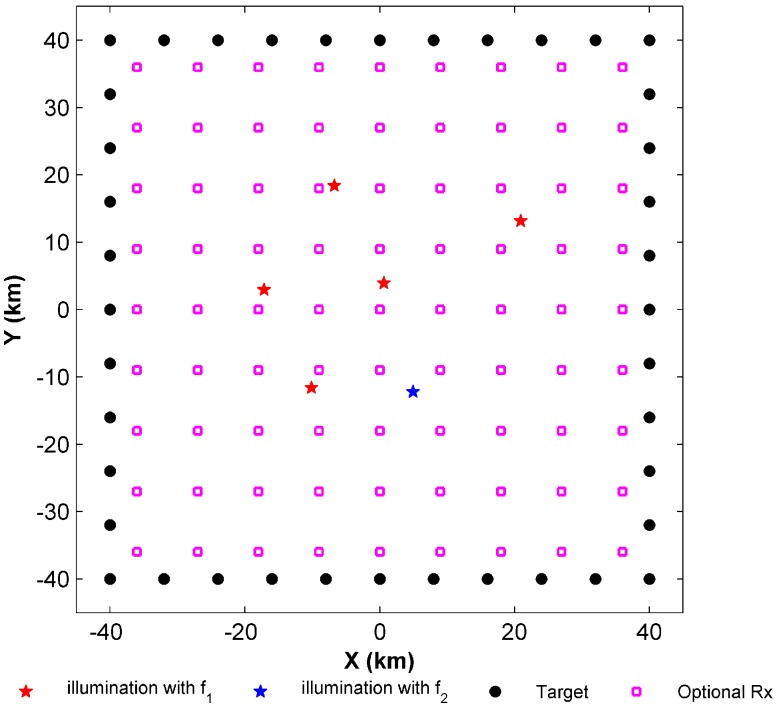
Simulation scenario.

Two-dimensional simulations are conducted, as shown in Figure 2. The targets of interest are located in an area of 80 km by 80 km and they are marked by the black circles. In general, the target points should be obtained by meshing the area of interest. However the calculation amount increases rapidly. Therefore, we select the targets of interest dispersed on the boundary of the area of interest to reduce the computational load. Normally, if all the boundaries are covered, the entire area of interest is also covered. The optional receivers marked by pink squares in Figure 2 are dispersed within this area.

Two types of illuminators are present in the simulation scenario. Their different frequencies, effective isotropic radiated powers, locations, and basic parameters are given in Table 1. The red pentagrams represent the first kind of illuminator and the blue pentagram represents the second. In the simulation, the structure of the red frequency network is SFN, and we assume that $K_{1}=4$. Namely the target should be covered by at least four bistatic pairs at the first frequency. In the second frequency network, only one bistatic pair is required to detect the target such that $K_{2}=1$. Though the abovementioned basic parameters are different, the other system parameters of the two frequency networks are the same (Table 2).

### 5.1. The Solution of PSCP

In the simulation, the propagation loss of the free space is treated as an example. The matrix Θ can be calculated according to (4). In the first simulation, we compare the solving algorithm of the PSCP. There are six methods for solving the PSCP: (a) Solve (13) using the $l_{1}$ norm surrogate method; (b) Solve (13) using Algorithm 3; (c) Solve (13) using Algorithm 3 and Algorithm 4; (d) Solve (15) using the $l_{1}$ norm surrogate method; (e) Solve (15) using Algorithm 3; (f) Solve (15) using Algorithm 3 and Algorithm 4. In the simulation, the threshold is set as $w_{mid}=[w^{*}(1)+w^{*}(NJ)]/2$.

**Figure 3:**
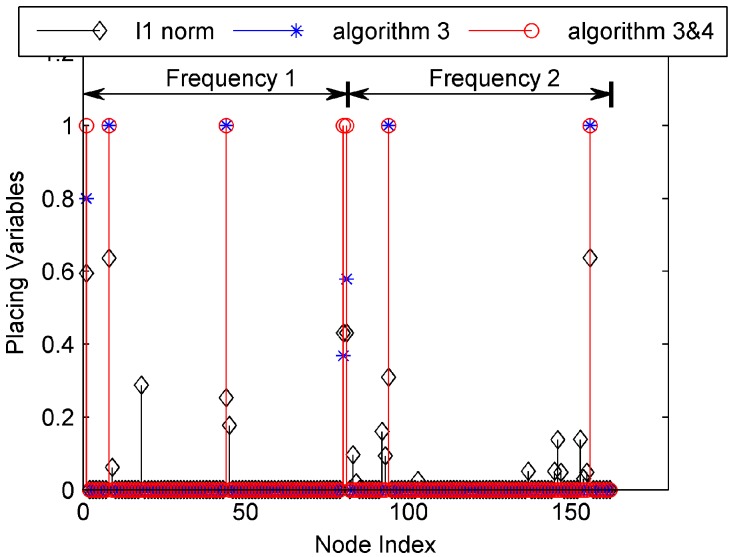
The solutions of (13) using three methods.

**Figure 4:**
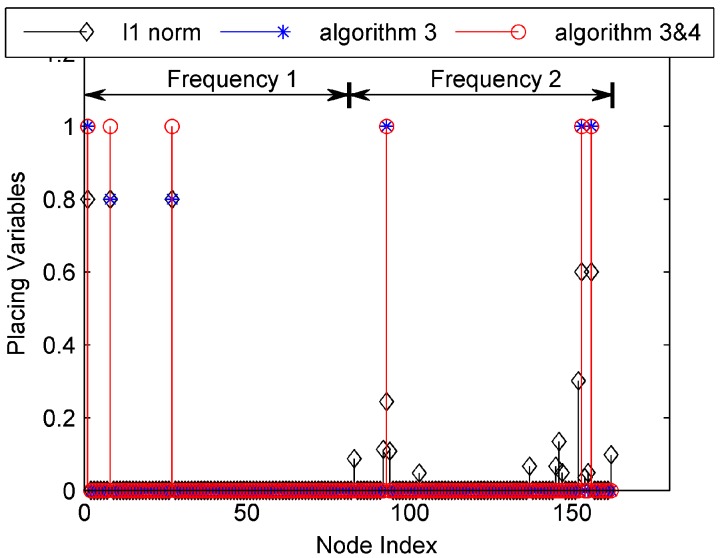
The solutions of (15) using three methods.

The solutions of the former three methods are shown in Figure 3, and the solutions of the latter three methods are shown in Figure 4. In the two figures, the first 81 indices represent the candidate receivers working with frequency 1, and the latter 81 indices represent those working with frequency 2. As expected, the solution of the reweighted $l_{1}$ minimization method has less nonzero placement variables than the solution of the $l_{1}$ norm surrogate method. The non-zero placement variables of the reweighted $l_{1}$ minimization method are sparse. When Algorithm 4 is used, the nonzero placement variables of the solution are reduced, which is a feasible solution of the PSCP.

In addition, the solution for (15) has less non-zero placement variables than that for (13), which indicates that the solution for (15) is more likely to be the optimal solution of the PSCP. In the simulation, $w_{mid}=7.97{\;\mathrm{dBm}}^{2}$. The objective function values can be obtained when these two solutions are returned back into (10). The objective function value of (13) is $7.94{\;\mathrm{dBm}}^{2}$, while the other is $7.29{\;\mathrm{dBm}}^{2}$. The model in (13) requires seven receivers, though its objective function value is larger than (15), thereby validating the suitability of (15) for solving the PSCP as compared to (13).

**Figure 5:**
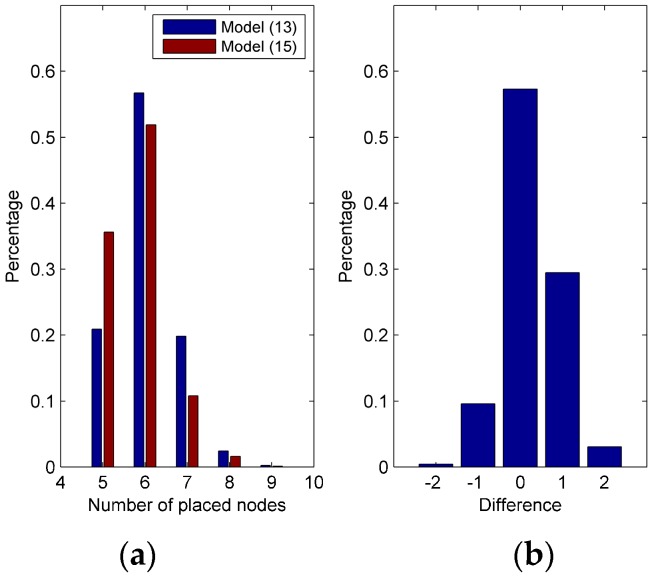
The MC simulation results with randomly generated candidate transmitters. (**a**) Distribution of the solutions of (13) and (15); (**b**) Distribution of the difference of the placed node number between two models.

MC simulations are conducted to test the adaptability of the model for solving the PSCP, wherein the candidate transmitters are randomly generated for each MC simulation. The other system parameters are shown in Table 1 and Table 2. A thousand MC simulations are conducted and $w_{mid}$ is set as the average of the upper and lower bounds. The MC simulation results are shown in Figure 5. Figure 5a shows the solution distribution for both (13) and (15). 87.5% of the solutions have five or six placed nodes using (15). The probability of acquiring five placed nodes using (15) is higher than for (13). A comparison between these two models is presented in Figure 5b. Of the solutions from (15), 57.6% are equal to those in (13), and 32.6% of the solutions from (15) are better than those from (13). The remaining 9.8% of the solutions exhibit the opposite result. Thus (15) is more suitable for statistically solving the PSCP. Since (15) is only statistically better, two models should be used to solve the PSCP, of which the better solution will be deemed acceptable

### 5.2. The solution of PPCP

**Figure 6:**
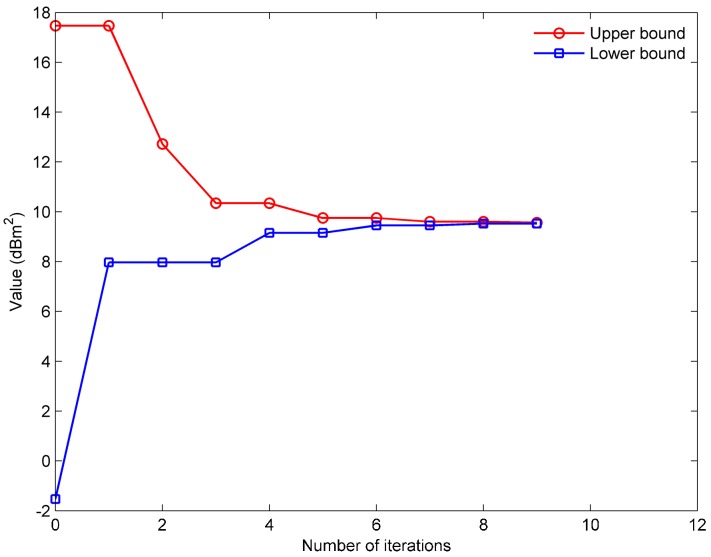
The curves of the upper and lower bounds vary with iteration times.

The performance of the algorithm for solving the PPCP is further investigated based on the solution of the PSCP. The simulation scenario is shown in Figure 2. Assuming that four receivers are placed, the implementation process for Algorithm 1 is demonstrated in Figure 6.

**Figure 7:**
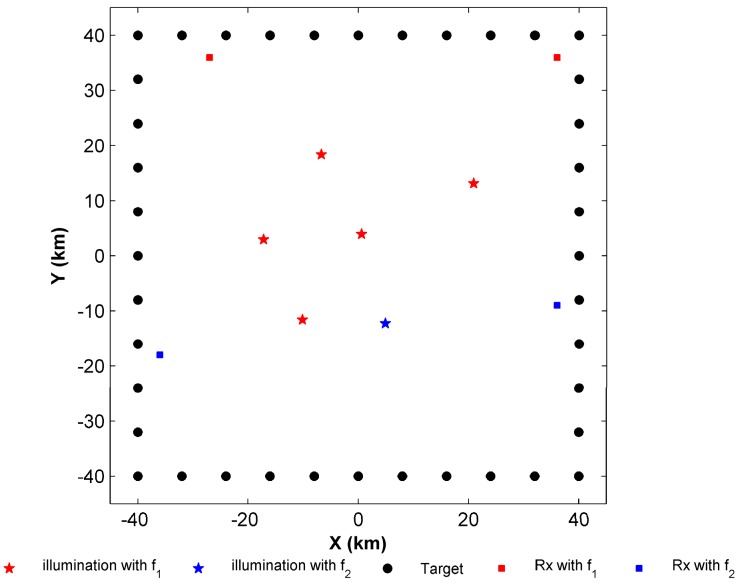
The optimal solution of the PPCP.

The upper and lower bounds are approximating each other to obtain the final optimal objective function value. In this simulation, $w^{*}(4)=9.56{\;\mathrm{dBm}}^{2}$. The optimal configuration marked by color-filled squares is illustrated in Figure 7. The red-filled squares represent the receivers that work with the first frequency and the blue-filled squares indicate the receivers using the second frequency.

The contrast between the existing algorithms and the proposed algorithm is analyzed in this paper. The comparative indicator is the curve of the objective function value changing with the number of iterations. The comparison algorithms in the simulation include the genetic algorithm (GA) [36] and the SA. The comparison can be divided into four types: Type 1, GA with initial population 1; Type 2, GA with initial population 2; Type 3, SA with initial value 1; Type 4, SA with initial value 2. The initial values and the initial populations are randomly generated.

**Figure 8:**
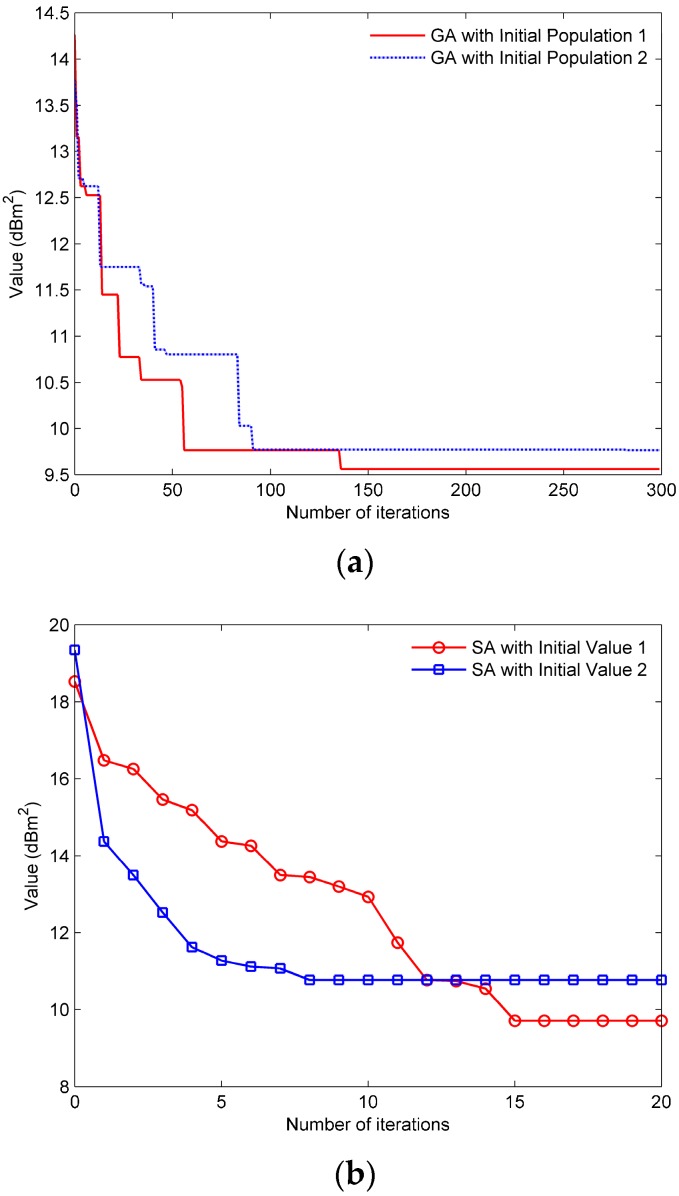
The curve of the objective function value changing with the number of iterations. (**a**) The result of GA with different initial population; (**b**) The result of SA with different initial value.

Some representative results are selected to demonstrate in Figure 8. Figure 8a demonstrates that the GA can be able to achieve the optimal solution after several iterations. However, it is time consuming and the probability of achieving the optimal solution is less than 10%. Figure 8b demonstrates some near-optimal solutions of the SA, namely the SA fails to obtain the global optimal solution in 500 simulations. The reason for this result is the premature convergence of the SA.

The computational complexity of different algorithms is analyzed in Section 4.5. In this simulation, we set to $K_{2i}=6$ and $K_{3i,\max}=10$. The simulation platform is a core i5-2410 computer with a 2.3 GHz main frequency. The average time consuming for a simulation of the three algorithms is given in Table 3. The SA obviously is the least time consuming, but its worst performance of achieving the optimal solution is the shown in Figure 8b. Algorithm 1 is slightly better than GA in term of time consuming, however, its performance of achieving the optimal solution is greatly improved because its probability of achieving the optimal solution is 1 in the simulation.

A final simulation is performed to test the capability for finding the global optimum of Algorithm 1. Four simulation scenarios similar to Figure 2 are present in the simulation. In each simulation scenario, different P and K values are considered. The comparison algorithm in the simulation included the GA and the SA. Both the GA and SA perform a hundred MC simulations. In each MC simulation, the maximum iteration is set to 300 times. The simulation results are shown in Table 4. In Table 4, all the simulations achieve the global optimal solution using Algorithm 1, thereby deeming Algorithm 1 suitable for achieving the optimal solution with Probability 1. The GA and SA may not be able to achieve the optimal solution, as most scenarios have very low probability of achieving the optimal solution. In term of computation, Algorithm 1 is less time consuming than 100 reiterations for GA and SA. Therefore, we can conclude that Algorithm 1 performs better in solving the PPCP than GA and SA. Nevertheless, future research requires more simulations to test the performance of the proposed algorithm in finding the global optimum.

### 5.3. Discussion

The following conclusions can be obtained from the numerical simulation.

(1) Algorithm 1 offers fast, polynomial approximation iteration strategies to solve the PPCP. The scope of the objective function value of the PPCP can be determined with only a few iterations. The iterations number depends on the required accuracy ε and the characteristic of the objective function. As a result, Algorithm 1 can not only provide the exact solution for PPCP, but also produce quite a few suboptimal solutions in the rapid decision-making process.
(2) The robustness of Algorithm 3 needs to be enhanced. The reason is that the reliability of the algorithm is closely related to the approximation of the convex relaxation model. In the process of the model conversion, the approximation degree of the convex relaxation model may be affected by the multiband network topology, the transmit power distribution of the multiband PRN, the propagation prediction accuracy and many other aspects. Fortunately, Algorithm 3 exhibits sufficient robustness in this simulation while solving both two conversion models. However, the theoretical performance analysis of the model (13) and (15) requires further research. In addition, the existence of a more suitable convex relaxation method for PSCP is worth being paid attention to.
(3) It is worth pointing out that the iterative reliability of Algorithm 1 relies on the accuracy of the PSCP solution, such that we have to solve both the model (11) and (13). Under such operation, Algorithm 1 almost invariably obtain the optimal solution in the present finite simulation. In terms of the time consuming and the performance of finding the optimal solution, Algorithm 1 has a strong advantage over the GA and SA.

## 6. Conclusions

Our research is driven by the practical application of the PRN. Two basic problems in constructing PRN are discussed in this paper: the placement of receivers and the selection of illuminators, from which a joint optimization model is established. To the best of the authors’ knowledge, the established model has not been reported in literature. In the model, a bisection algorithm is proposed to solve the PPCP. Simulation results indicate the effectiveness of the proposed algorithm. Future work must consider receivers with different costs. Moreover, improvement in the PPCP model based on the actual situations is also a valuable research direction.

## Figures and Tables

**Table 1:** The Basic Parameters of Two Frequency Networks.

The Red Illuminator		The Blue Illuminator	
Parameters	Values	Parameters	Values
Frequency	600 MHz	Frequency	650 MHz
Bandwidth	8 MHz	Bandwidth	10 MHz
Power	1 kW	Power	2 kW

**Table 2:** The Other System Parameters of Two Frequency Networks.

Parameters	Values
Coherent integration time	0.1 s
Minimum detection SNR	12 dB
The number of antenna arrays	8
Hardware system loss	6 dB
Noise factor	5 dB
Reference temperature	290 K

**Table 3:** The Average Time Consuming of the Three Algorithms.

Algorithms	Average Time Consumption
Algorithm 1	165.1 s
GA	192.8 s
SA	8.4 s

**Table 4:** Simulation Results Obtained With Algorithm 1, GA, and SA for PPCP.

Scenario	*P*	*K*	Algorithm 1	GA		SA	
				Optimal Value	Probability	Optimal Value	Probability
1	4	[4 1]	8.44	8.44	0.10	8.65	0.02
		[1 1]	7.42	7.42	0.13	7.42	0.06
	5	[4 1]	7.86	8.18	0.47	7.86	0.05
		[1 1]	6.77	6.77	0.13	6.77	0.27
	6	[4 1]	6.72	6.72	0.02	6.72	0.15
		[1 1]	5.92	5.92	0.10	5.92	0.36
2	4	[4 1]	8.40	8.40	0.22	8.78	0.68
		[1 1]	7.61	7.61	0.22	7.61	0.80
	5	[4 1]	7.97	7.97	0.41	7.97	0.14
		[1 1]	7.10	7.10	0.27	7.10	0.80
	6	[4 1]	6.94	6.94	0.01	6.94	0.13
		[1 1]	5.54	5.54	0.06	5.54	0.55
3	4	[4 1]	8.88	8.88	0.63	8.88	0.10
		[1 1]	7.64	7.64	0.17	7.64	0.08
	5	[4 1]	8.82	8.82	0.68	8.82	0.35
		[1 1]	7.43	7.43	0.41	7.43	0.49
	6	[4 1]	5.85	6.74	0.01	6.74	0.06
		[1 1]	5.84	5.84	0.01	5.84	0.13
4	4	[4 1]	9.12	9.12	0.88	9.12	0.68
		[1 1]	6.91	6.91	0.17	6.91	0.07
	5	[4 1]	7.67	7.67	0.03	7.67	0.08
		[1 1]	5.51	5.68	0.04	5.61	0.24
	6	[4 1]	6.91	6.91	0.05	6.91	0.08
		[1 1]	4.89	4.89	0.01	4.89	0.15

**Figure A1:**
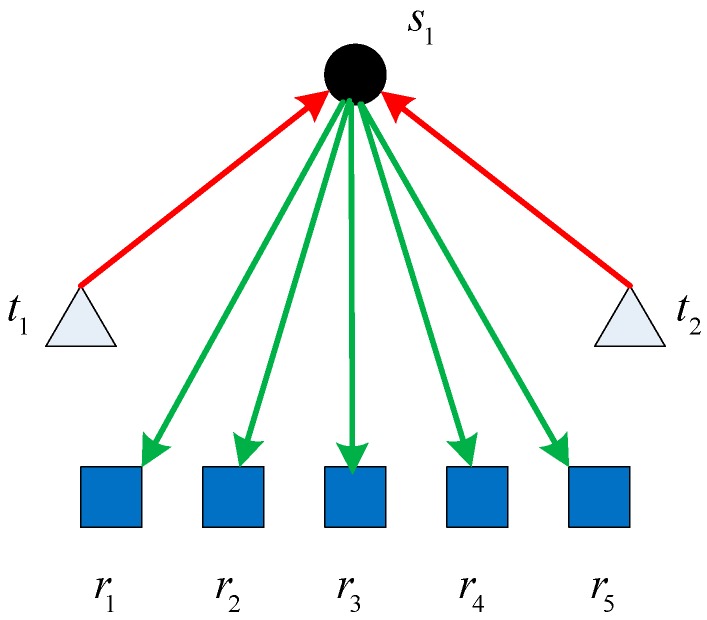
An example of a special network topology diagram.

## Appendix A. A Special Network Topology

We describe a very special network topology for taking the equality mark as follows. If $w^{*}(p)=w^{*}(p+1)$, then $w^{*}(p)=w(p+1|p)=w^{*}(p+1)$. This equation indicates that the optimal objective function value will be unchanged following the addition of a receiver, such that the new configuration will also produce an optimal configuration.

In Figure A1, the target $s_{1}$ and the receiver $r_{3}$ lie on the perpendicular bisector of the two transmitters, and the perpendicular bisector symmetrical point of $r_{1}$ and $r_{2}$ are $r_{5}$ and $r_{4}$ respectively. In this special topology, $r_{3}$ was determined as the optimal placement. Therefore, it can be concluded that $w^{*}(1)=w^{*}(2)=\cdots =w^{*}(5)$.

We further assume that the presence of two symmetric targets to deduce $w^{*}(2)=w^{*}(3)=\cdots =w^{*}(5)$. The possibility of the inequality taking mark of the quality is minimized with a rise in the number of the targets. Since symmetry in the network topology is necessary, it is difficult to produce this special network topology in practice.
